# A Nomogram for Predicting ADHD and ASD in Child and Adolescent Mental Health Services (CAMHS)

**DOI:** 10.3390/jcm13082397

**Published:** 2024-04-19

**Authors:** Hilario Blasco-Fontecilla, Chao Li, Miguel Vizcaino, Roberto Fernández-Fernández, Ana Royuela, Marcos Bella-Fernández

**Affiliations:** 1 Instituto de Investigación, Transferencia e Innovación, Ciencias de la Saludy Escuela de Doctorado, Universidad Internacional de La Rioja, 26006 Logroño, Spain; 2 Center of Biomedical Network Research on Mental Health (CIBERSAM), Carlos III Institute of Health, 28029 Madrid, Spain; 3 Faculty of Medicine, Universidad Autónoma de Madrid, 28049 Madrid, Spain; ericlimed@gmail.com; 4 Centro de Salud San Carlos, 28200 El Escorial, Spain; vizcainodasilva@gmail.com; 5 Hospital Universitario Infanta Cristina, 28981 Madrid, Spain; rffernandez@salud.madrid.org; 6 Biostatistics Unit, Hospital Universitario Puerta de Hierro Majadahonda, 28222 Majadahonda, Spain; aroyuela@idiphim.org; 7 Puerta de Hierro University Hospital, 28222 Majadahonda, Spain; marcosbellafernandez@gmail.com; 8 Faculty of Psychology, Universidad Autónoma de Madrid, 28049 Madrid, Spain

**Keywords:** ADHD, ASD, predictive models, nomograms

## Abstract

**Objectives:** To enhance the early detection of Attention Deficit/Hyperactivity Disorder (ADHD) and Autism Spectrum Disorder (ASD) by leveraging clinical variables collected at child and adolescent mental health services (CAMHS). **Methods:** This study included children diagnosed with ADHD and/or ASD (*n* = 857). Three logistic regression models were developed to predict the presence of ADHD, its subtypes, and ASD. The analysis began with univariate logistic regression, followed by a multicollinearity diagnostic. A backward logistic regression selection strategy was then employed to retain variables with *p* < 0.05. Ethical approval was obtained from the local ethics committee. The models’ internal validity was evaluated based on their calibration and discriminative abilities. **Results:** The study produced models that are well-calibrated and validated for predicting ADHD (incorporating variables such as physical activity, history of bone fractures, and admissions to pediatric/psychiatric services) and ASD (including disability, gender, special education needs, and Axis V diagnoses, among others). **Conclusions:** Clinical variables can play a significant role in enhancing the early identification of ADHD and ASD.

## 1. Introduction

Attention Deficit/Hyperactivity Disorder (ADHD) and autism spectrum disorder (ASD) are two of the most prevalent neurodevelopmental disorders among children [1,2,3]. Much research has been devoted to exploring the risk and protective factors for ADHD [4,5,6] and ASD [7,8]. Unfortunately, predicting ADHD and ASD based on available clinical information, especially at an early age, presents significant challenges. The difficulty in early identification of both ADHD and ASD lies in the current reliance on clinical, subjective data for diagnosing mental disorders. This data depends heavily on the observer’s perspective and experience, making the process inherently subjective. In other words, the challenge of achieving an accurate diagnosis is compounded by the lack of biomarkers for these mental disorders. Furthermore, complicating matters is the fact that the manifestations of these disorders vary widely among patients [9]. Furthermore, patients with either ADHD or ASD frequently show comorbidities with other mental disorders. These difficulties make finding potential biomarkers or clinical indicators especially important.

For a biomarker to be clinically useful, it must have high sensitivity (>90%) and specificity (>90%) [10]. Moreover, considering the significant genetic component of both disorders, parents may not always accurately convey their children’s clinical manifestations. They tend to normalize certain symptoms that they, too, have experienced. Against this backdrop, the integration of new tools to enhance the early detection of ADHD and ASD is exceedingly justified.

Only in recent times have predictive models for ADHD or ASD been introduced. The majority of these models leverage artificial intelligence. For instance, Lee et al. [11] proposed a model for ADHD prediction using negative emotionality, communication abilities, coarse motor skills, social competence, and academic performance as predictors. Tachmazidis et al. [12] used a hybrid machine-learning/expert system approach to develop their ADHD predictive model using items from tests of ADHD, drug and alcohol abuse, and personality, mood and anxiety disorders as inputs. Slobodin et al. [13] also used machine learning to build predictive models from variables of the CPT. Sen et al. [14] used data from magnetic resonance imaging to build their predictive model. Maniruzzaman et al. [15] and Garcia-Argibay et al. [16] performed a variety of machine-learning-based methods using clinical variables as predictors. Only these two last studies, as well as Silverstein et al. [17] and Caye et al. [18], used a regression-based approach for their predictive model. Interestingly, however, Caye et al. [18] used both logistic regressions and machine-learning approaches, and they found that machine learning did not outperform logistic regressions. Regarding ASD, some predictive models have been proposed based on neurobiological markers [14,19,20] or screening tests [21]. However, despite their potential utility, these models—especially those utilizing machine-learning techniques—suffer from a lack of transparency. The variables they incorporate and their respective significance within the models remain obscure. A notable exception to this pattern is the model proposed by Caye et al. [18]; these authors have even created a practical calculator for estimating the risk of ADHD, which is readily accessible.

The primary objective of this study is to identify, from the clinical variables commonly reviewed in patient charts at Child and Adolescent Mental Health Services (CAMHS), those that can more reliably predict a diagnosis of ADHD and ASD. This could facilitate the development of predictive models for ADHD and/or ASD using medical information that is readily available. With these clinical variables, the ultimate aim of this study is to develop a series of nomograms (refer to the Section 2 for a detailed description of nomograms and their application), which could be utilized as a calculator in a manner similar to that developed by Caye et al. [18]. The distinctions between their research and our current study lie in the sample population (they utilized adult samples, whereas we employ data from children and adolescents) and the number and variety of clinical variables examined.

## 2. Methods

### 2.1. Participants

Data were gathered from children who attended the Child and Adolescent Mental Health Services (CAMHS) at Hospital Universitario Puerta de Hierro Majadahonda. A retrospective evaluation was conducted on a sample size of *n* = 857 patients. The study included children diagnosed with ADHD and/or ASD, with the only exclusion criterion being the absence of an ADHD or ASD diagnosis. Information was extracted from the patients’ clinical records, and the database was anonymized prior to any analysis. Accordingly, we have incorporated some information about anonymization. We used dissociated databases and followed the standard Ethics permitted by the Ethical Committee of the Hospital Universitario Puerta de Hierro Majadahonda to proceed with the study. The study was approved by the local ethics committee (PI 112_17, 11 September 2017).

### 2.2. Outcome Variables

The outcomes used in the three logistic models were: primary ADHD, ADHD subtype (only for those patients diagnosed with ADHD), and primary ASD. A single child psychiatrist clinically performed these diagnoses including the five axes of the Diagnostic and Statistical Manual, Fourth version [22] criteria. Axis I diagnosis was made using the fifth version [9]. Dichotomous medical and clinical variables were coded as “1” if the patient had the disorder or condition and “0” if the patient did not have the disorder. ADHD subtype was coded as “1” if the subtype was Hyperactive or Combined and “0” if the subtype was Inattentive.

### 2.3. Potential Predictors

The predictor variables are described in Table 1. Data were gathered from children who attended the CAMHS at Hospital Universitario Puerta de Hierro Majadahonda. A retrospective evaluation was conducted on a sample size of *n* = 857 patients. The study included children diagnosed with ADHD and/or ASD, with the only exclusion criterion being the absence of an ADHD or ASD diagnosis. Information was extracted from the patients’ clinical records, and the database was anonymized prior to any analysis.

### 2.4. Statistical Analyses

The goal was to construct three logistic regression models to predict ADHD as a primary diagnosis, the ADHD subtype (either inattentive or combined/hyperactive) exclusively in patients diagnosed with ADHD or ASD as a primary diagnosis, respectively. Univariate logistic regression analyses were conducted as an initial step to select variables whose regression coefficients achieved statistical significance and those of clinical importance. Subsequently, a multicollinearity diagnostic among the chosen variables was performed using condition numbers [23] and the Variance Inflation Factor (VIF). Once the variables were selected, we performed a backward logistic regressions selection strategy, removing the variable with the higher *p*-value in every step, to include in each final model only those variables with *p* < 0.05. For each final model, the internal validation was evaluated based on the calibration and discrimination abilities.

A model is considered calibrated when its predictions of the proportion or number of cases (predicted risk of outcome) align closely with the observed proportion of cases (observed risk of outcome). To evaluate model calibration, linear regressions were performed on the predicted versus observed risk of outcome, with their slopes serving as measures of calibration. The closer this slope is to 1, the better the model’s calibration. Additionally, we examined calibration-in-the-large (CITL), which compares the average of all predicted risks to the mean observed risk. This parameter reflects whether predictions are systematically too low (CITL < 0) or too high (CITL > 0), with values near 0 indicating good calibration. Discriminability of a model refers to its ability to accurately classify participants as having or not having the outcome—that is, participants with the outcome are predicted to have it, and vice versa. The C-statistic was used to assess discriminability [24], equivalent to the area under the ROC curve.

Both abilities were assessed using a bootstrap resample approach, through the “bsvalidation” command from STATA [25].

To improve the model interpretation, we developed a nomogram for each one of the models.

All analyses were performed with STATA, version 17.0 (College Station, TX, USA, April 2021) and R, version 4.1.2 (R Development Core Team, Vienna, Austria, November 2021). The R package “rms” [26] was used to estimate the logistic regression models and draw the nomograms, and the R package “multiColl” [27] was used to calculate the condition numbers.

### 2.5. Graphical Outcomes: Nomograms

A nomogram is a graphical tool used to interpret a pre-calculated model and its outcomes based on a specific set of predictor variable values. In this context, the model in question is a logistic regression model, predicting the likelihood of having ADHD (nomogram 1), a specific ADHD subtype (nomogram 2), or ASD (nomogram 3).

A nomogram features an upper horizontal “Points” line with a points scale. This scale is designed to convert the scores of each variable into a unified metric. Directly beneath this scale, horizontal lines represent each predictor in the model, each with its unique metric based on the potential values of the predictor. To translate a score from its original metric to the unified metric, one must locate the raw score on its respective line and draw a vertical line up to the “Points” line; the intersection point indicates the score in the unified metric. Summing up these scores for all variables yields a total score.

The nomogram’s final two lines facilitate the conversion of this total score into a probability. This is done by locating the total score on the “Total points” line and drawing a vertical line down to intersect with the final line, where the estimated probability can be read. To illustrate, consider two hypothetical examples based on a fabricated model predicting ADHD, assuming it is influenced by three variables: Gender, adoption status, and age at first words. These examples demonstrate the application of the nomogram to predict ADHD using this model.

**Figure 1:**
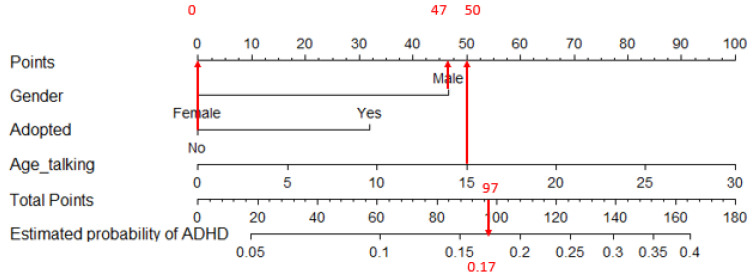
Sample nomogram for applying the hypothetical model to a male patient who was not adopted and began speaking at 15 months. Notice the arrows linking the variable values under “Gender”, “Adopted”, and “Age_talking” to the upper horizontal line, yielding their respective partial scores (47, 0, and 50). The total score is the sum of these partial scores: 47 + 0 + 50 = 97. This total score is then transformed into a probability using the two bottom horizontal lines. In this scenario, the estimated probability of having ADHD is 0.17.

Example 1: For a male patient who was not adopted and began speaking at 15 months, as per the fabricated model, being male contributes 47 points, not being adopted adds 0 points, and starting to speak at 15 months adds 50 points to the total score, summing to 97 points. This total score translates into a 0.17 probability of having ADHD. Accordingly, the fictitious model and its nomogram estimate a 0.17 probability of ADHD for this patient. Figure 1 shows the nomogram of this hypothetical example.

**Figure 2:**
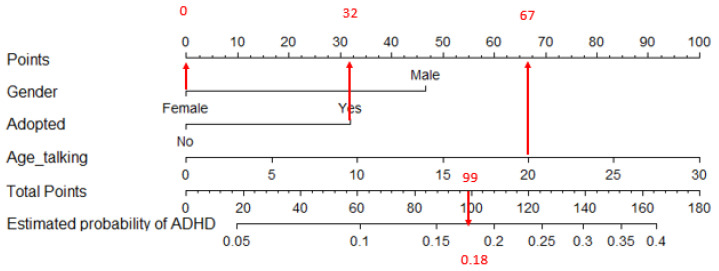
Nomogram associated with applying the made-up model to a female patient who was adopted and started talking at the age of 20 months. Observe the arrows connecting the variable values in “Gender”, “Adopted”, and “Age_talking” with the upper horizontal line to obtain their respective partial scores (0, 32, and 67). The total score is the sum of the partial scores; 0 + 32 + 67 = 99. This total score is converted into a probability using the two lower horizontal lines. In this case, the estimated probability of having ADHD is 0.18.

Figure 2 utilizes the same hypothetical predictive model to estimate the probability of a female patient, who was adopted and began speaking at 20 months, having ADHD. Being female contributes 0 points to the score. Being adopted adds 32 points, and starting to speak at the age of 20 months contributes 67 points. Thus, the total score amounts to 0 + 32 + 67 = 99 points. This total score is subsequently converted into a probability of 0.18 for this patient having ADHD.

It is noteworthy that the predictive power of each variable is reflected in the length of their corresponding lines in the nomogram. Variables with a greater predictive capability will have larger lines than those with a lower predictive capability. The variable weighting is also reflected in the score which a certain variable may give relative to the total amount of points.

## 3. Results

Table 2 presents the distribution of sociodemographic and clinical variables in the sample. Overall, we developed three logistic models with satisfactory predictive performance. Table 3 displays the models generated for predicting ADHD, its subtypes, and ASD.

The model for ADHD prediction shows good calibration and discrimination power, and no multicollinearity was detected. The predictive equation was: ADHD = −1.340 + 0.752 × (Physically active) + 0.697 × (History of bone fractures) − 0.034 (Age of first spoken word, in months) − 0.083 × (Disability) − 0.831 × (Pediatric admission) + 0.612 (Risky pregnancy) − 1.079 × (Urine control) + 1.936 × (Fecal control) − 1.243 × (Special education needs) + 1.216 × (Medical treatment) − 1.432 × (Psychiatric admission) + 1.220 (Comorbidity with another Axis I diagnosis). The slope of the calibration plot was 0.863, the CITL was 0.031, and the C-statistic was 0.817.

The model to predict ADHD subtype from patients diagnosed with ADHD did not show multicollinearity. The predictive equation was: Probability of Hyperactive/Combined ADHD = 2.396 + 0.554 × (History of bone fractures) + 1.392 × (Psychiatric admission) + 0.580 × (Male) − 0.150 × (Age in years). The slope of the calibration plot was 0.872, the CITL was 0.004, and the C-statistic was 0.663.

Finally, the model for primary diagnosis of ASD did not show multicollinearity. The predictive equation for ASD was: ASD = −0.286 + 1.124 × (Special education needs) − 1.053 × (History of bone fractures) + 2.330 × (Disability) + 1.299 × (Male) − 0.038 × (Diagnostic in Axis V). The slope of the calibration plot was 0.861, the CITL was 0.008, and the C-statistic was 0.894.

**Figure 3:**
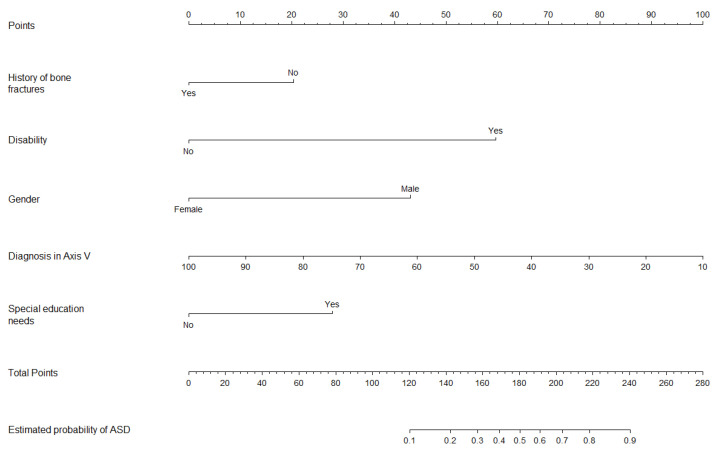
Nomogram for ADHD prediction model.

**Figure 4:**
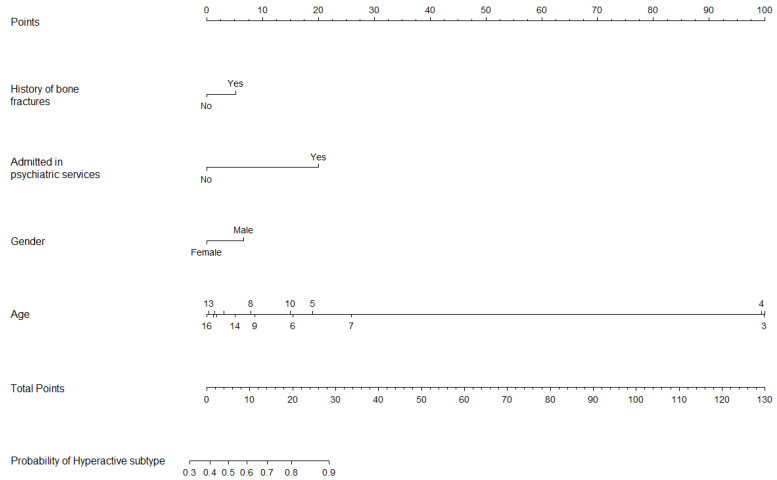
Nomogram for ADHD subtype prediction model.

**Figure 5:**
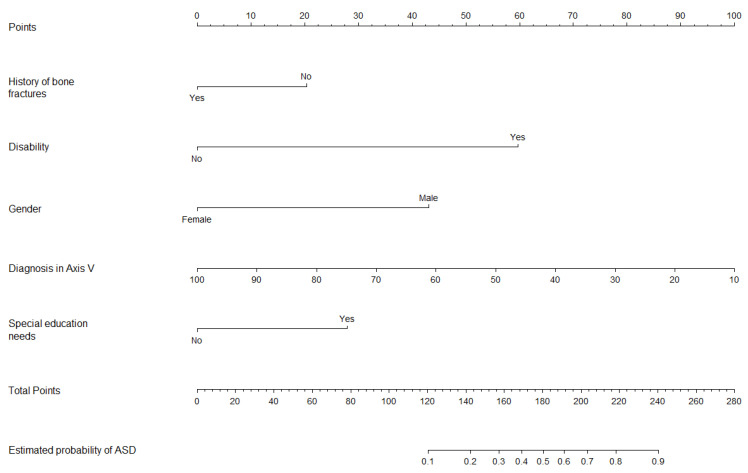
Nomogram for ASD prediction model.

Figure 3, Figure 4 and Figure 5 show the nomograms of primary ADHD model, ADHD subtype, and ASD, respectively.

## 4. Discussion

In this study, we aimed to create predictive models for ADHD and ASD using variables commonly gathered during routine pediatric or psychiatric assessments. We successfully developed models with strong predictive capabilities for a primary diagnosis of either ADHD or ASD. However, the model for predicting ADHD subtypes (either inattentive or hyperactive/combined) did not perform adequately.

The primary objective was to offer clinical practitioners a quick and useful tool for estimating predictions for two of the most prevalent neurodevelopmental disorders. Our methodology is akin to that of Caye et al. [18]. However, they developed their calculator based on data from adult patients, whereas our tool is designed for use in child and adolescent psychiatry services. Moreover, our predictive models incorporate a wide range of variables that, nevertheless, an average practitioner would typically have at their disposal simply by conducting a standard review of their patients’ clinical records.

### 4.1. Predictors of ADHD

Numerous variables were incorporated into the final model for diagnosing ADHD. Initially, a risky pregnancy was reported almost twice as often in children diagnosed with ADHD. Various researchers have highlighted the association between ADHD in offspring and several risk factors during pregnancy, including early pregnancy or pregnancy-induced hypertension [28]. Additionally, we discovered that children with ADHD were three times more likely to experience delays in achieving urinary control, aligning with extensive literature suggesting that enuresis is a predictor of ADHD [29]. For example, in a study assessing the prevalence of ADHD among 86 children with enuresis, the authors found that the likelihood of a child with ADHD experiencing voiding issues after 2 years of follow-up was approximately 3.17 times higher compared to children without ADHD [30]. More challenging to elucidate is the negative association we observed between delayed fecal control and ADHD. Our findings appear to contradict existing evidence suggesting a positive relationship between fecal incontinence and ADHD [29,31,32]. However, our results are similar to those of a study that found an association between enuresis, but not encopresis, and ADHD [33]. Moreover, these findings could be attributed to the relatively high average age (11.6 ± 3.5) of the patients assessed in this study at the time of their psychiatric consultation. At this age, the prevalence of encopresis is typically low. For instance, in a population-based study involving around 20,000 children in Amsterdam, the prevalence of encopresis was found to be 4.1% among children aged 5 to 6 years, and 1.6% in those aged 11 to 12 years. In our sample, the prevalence of encopresis was 3% in the group with ADHD and 17.8% in the group without ADHD.

Furthermore, two predictors of an ADHD diagnosis were associated with physical activity: (1) increased physical activity, which is indeed a criterion of hyperactivity and, therefore, may be considered a stronger clinical marker of ADHD; and (2) an elevated risk of bone fractures. The prevalence of bone fractures among children and adolescents diagnosed with ADHD, as reported in a recent meta-analysis, was 4.83% (95% CI: 3.07–6.58%) [34]. Furthermore, our finding is in keeping with several studies demonstrating a higher risk of bone fractures among patients diagnosed with ADHD [35,36,37,38]. The same studies also present some conflicting data regarding the risk of stress fractures, as the use of methylphenidate has been linked to adverse effects on bone mass.

Lastly, it is unsurprising that the presence of comorbidities in Axis I was predictive of an ADHD diagnosis, given that ADHD often co-occurs with other conditions such as ASD or learning disabilities [39].

### 4.2. Predictors of ADHD, Hyperactive/Combined Subtype

The model predicting ADHD subtypes included fewer variables and exhibited low predictive capabilities. Nonetheless, certain variables slightly enhanced its predictive power. These findings indicate that female children are more likely to be diagnosed with the Inattentive subtype of ADHD, whereas male children are more often diagnosed with Hyperactive or Combined subtypes, aligning with previous research [40]. Moreover, a history of bone fractures was more closely associated with the ADHD-hyperactive/combined subtype. Surprisingly, much of the research exploring the relationship between ADHD and bone fractures (or the broader concept of traumatic injuries) did not take into account the potential influence of ADHD subtypes. Nonetheless, several authors have noted an elevated risk of accidental injuries among ADHD populations, regardless of subtype [41,42]. On the other hand, at least one study reported that traumatic dental injury is more frequently reported among the hyperactive subtype [43]. Lastly, psychiatric admission also emerged as a predictor for the hyperactive/combined subtype of ADHD. One plausible explanation is that this subtype is often linked to disruptive behavior, which in turn is associated with a higher risk of psychiatric hospitalization [44].

### 4.3. Predictors of ASD

The model of ASD prediction reflects a bias towards male gender consistent with previous literature [45,46]. However, the most significant factors predicting an ASD diagnosis were disability and special education needs. ASD is highly heterogeneous and often co-occurs with intellectual disabilities. Indeed, the overlap between ASD and intellectual disability has complicated both the diagnosis and research into the genetic factors associated with autism [47,48]. The relationship between autism and special education needs is particularly noteworthy. Indeed, a substantial proportion of children with ASD are enrolled in special education programs [49,50]. Lastly, a history of bone traumas was negatively associated with ASD compared to other psychiatric disorders, such as ADHD. While existing literature indicates that ASD is also linked with an increased risk of trauma, our perspective aligns with the findings of Diguiseppi et al. [51], who showed that the relationship between ASD and trauma was mediated by attention problems. Consequently, the prevalence of bone trauma may assist in distinguishing between children with ASD and those with ADHD.

### 4.4. Strengths and Limitations

The primary strength of this study lies in the ability to predict the presence of ADHD or ASD using variables that are readily accessible in clinical settings. Additionally, the substantial sample size ensures a degree of representativeness among child and adolescent psychiatric patients. Moreover, employing a practical tool like a nomogram enables clinicians and practitioners to easily implement the models introduced in this study.

However, this study has several limitations. Firstly, all patients were recruited and assessed by a single professional (the principal investigator, HBF), limiting the generalizability of our findings to other CAMHS settings, despite these results aligning with existing literature. Secondly, clinical data utilized the DSM-IV’s five axes instead of the DSM-5 classification, reflecting the principal investigator’s preference for the DSM-IV’s comprehensive multiaxial approach over the DSM-5’s. This choice, however, means the study relied on somewhat outdated information regarding ADHD. Future studies should consider using the DSM-5. Thirdly, the study’s findings are based on clinical data collection rather than scales, which underscores the study’s unique appeal in enabling the early identification of children at risk for ADHD without the need for scales. The clinical variables included were selected based on the principal investigator’s routine practice and experience, potentially omitting other relevant variables from the predictive models. Nonetheless, the nomograms provided align closely with scientific literature and can underpin screening diagnoses, particularly in settings where evaluation time is limited.

## 5. Conclusions

The models introduced in this paper reasonably predict the likelihood of a patient attending CAMHS having ADHD or ASD, based on clinically available variables. In summary, the models for predicting ADHD and ASD incorporate key variables that can aid practitioners in anticipating the occurrence of these disorders. However, further research is needed to improve discrimination between ADHD subtypes, potentially beyond the scope of chart review information.

## Figures and Tables

**Table 1:** Variables explored in this study.

Variable	Operationalization	Categories	Frequencies or Mean (sd) *
Age	How old (in years) is the patient?	Continuous variable	11.1 (3.9)
Gender	What is the gender of the patient?	Male (0) or Female (1)	Male = 593 Female = 276
Adopted	Was the child adopted?	Yes (1) or No (0)	No = 798 Yes = 52
Family (first grade) psychiatric antecedents	Does the patient have any first-grade relative formally diagnosed with any mental disorder?	Yes (1) or No (0)	No = 332 Yes = 471
Risky pregnancy	Was the patient’s gestation a risky pregnancy?	Yes (1) or No (0)	No = 608 Yes = 236
Use of toxic substances by the mother during pregnancy	Did the patient’s mother take any toxic substances during pregnancy?	Yes (1) or No (0)	No = 783 Yes = 18
Stress/depression during pregnancy	Did the patient’s mother suffer stress or depression during pregnancy?	Yes (1) or No (0)	No = 644 Yes = 192
Preeclampsia during pregnancy	Did the patient’s mother suffer preeclampsia during pregnancy?	Yes (1) or No (0)	No = 805 Yes = 23
Comorbidity in Axis I (Clinical Disorders)	Does the patient have a second Axis I diagnosis?	Yes (1) or No (0)	No = 245 Yes = 616
Diagnosis in Axis III	Does the patient have a diagnosis of a disorder included in Axis III (general medical condition)?		No = 59 Yes = 809
Atopy	Did the patient suffer atopy?	Yes (1) or No (0)	No = 485 Yes = 371
History of bone fractures or repetitive injuries evaluated or not at the ER?	Has the patient ever suffered a bone fracture? Has the patient had repetitive injuries evaluated at the ER?	Yes (1) or No (0)	No = 469 Yes = 378
Diagnosis in Axis IV	Does the patient have a diagnosis of a disorder included in Axis IV (psychosocial problems)?	Yes (1) or No (0)	No = 187 Yes = 661
Disability	Does the patient suffer any disability?	Yes (1) or No (0)	No = 717 Yes = 140
Urine control (day and evening)	Does the patient control his/her urine?	Yes (1) or No (0)	No = 112 Yes = 713
Fecal control	Does the patient control his/her feces?	Yes (1) or No (0)	No = 162 Yes = 761
Started walking	Age (in months) at which the patient started walking	Continuous	15.76 (8.35)
Special education needs	Does the patient have any special education needs?	Yes (1) or No (0)	No = 716 Yes = 108
Genetics	Any confirmed genetic disease?	Yes (1) or No (0)	No = 801 Yes = 43
Physically active	Does the patient exercise regularly?	Yes (1) or No (0)	No = 259 Yes = 573
Admitted to the psychiatric acute inpatient unit?	Has the patient ever been admitted to the psychiatric acute inpatient unit?	Yes (1) or No (0)	No = 794 Yes = 50
Admitted (hospitalization) in pediatric services	Has the patient ever been hospitalized in pediatric services?	Yes (1) or No (0)	No = 709 Yes = 130
Medical treatment	Is the patient taking any medication regarding a general medical condition?	Yes (1) or No (0)	No = 399 Yes = 461
Axis V score	Which is the global assessment scale? (0–100)	Continuous	68.98 (12.16)

* Please note that summing the frequencies of each variable gives different results due to incomplete clinical records.

**Table 2:** Sociodemographic variables.

	Total	ADHD (*n* = 599)	No ADHD (*n* = 246)	*p*	Hyperactive/Combined (*n* = 414)	Inattentive (*n* = 185)	*p*	ASD (*n* = 84)	No ASD (*n* = 84)	*p*
Age	11.1 (3.9)	11.6 (3.5) 3–18	9.8 (4.6) 1.5–22	<0.001	11.1 (3.5)	12.7 (3.0)	<0.001	8.6 (4.4)	11.3 (3.7)	<0.001
Sex (% Female)	31.7%	29.9%	35.4%	0.139	29.6%	39.5%	<0.001	11.9%	33.6%	<0.001
Nationality (% Spanish)	84.9%	85.0%	84.5%	0.9375	86.4%	84.4%	0.599	76.2%	85.8%	0.029

**Table 3:** Logistic regression model for ADHD, ADHD subtype, and ASD.

Model	Factor	OR (95% CI)	VIF	Condition Number
ADHD (*n* = 632)	Constant			11.68
	Risky pregnancy (No = 0, Yes = 1)	1.85 (1.14, 3.00)	1.063	
	Age of first words (in months)	0.86 (0.73, 1.02)	1.125	
	Urine control (No = 0, Yes = 1)	0.32 (0.13, 0.88)	1.630	
	Fecal control (No = 0, Yes = 1)	7.14 (2.56, 19.23)	1.623	
	Special educational needs (No = 0, Yes = 1)	0.29 (0.13, 0.63)	1.445	
	Disability (No = 0, Yes = 1)	0.34 (0.18, 0.67)	1.425	
	Physically active (No = 0, Yes = 1)	1.63 (1.05, 2.52)	1.052	
	History of bone fractures (No = 0, Yes = 1)	2.20 (1.44, 3.37)	1.036	
	Medical treatment (No = 0, Yes = 1)	3.33 (2.17, 5.05)	1.065	
	Pediatric admission (No = 0, Yes = 1)	0.44 (0.26, 0.74)	1.023	
	Psychiatric admission (No = 0, Yes = 1)	0.29 (0.12, 0.70)	1.023	
	Comorbidity with Axis I diagnose (No = 0, Yes = 1)	3.70 (2.32, 5.54)	1.070	
ADHD subtype: Hyperactive/Combined (*n* = 551)	Constant			2.79
	History of bone fractures (No = 0, Yes = 1)	1.66 (1.14, 2.54)	1.020	
	Psychiatric admission (No = 0, Yes = 1)	6.43 (1.36, 28.31)	1.007	
	Sex (Male = 0, Female = 1)	0.60 (0.41, 0.89)	1.058	
	Age (in years)	0.86 (0.81, 0.91)	2.896 *	
ASD (*n* = 634)	Constant			3.02
	Special educational needs (No = 0, Yes = 1)	2.78 (1.25, 6.20)	1.685	
	History of bone fractures (No = 0, Yes = 1)	0.47 (0.24, 0.93)	1.013	
	Disability (No = 0, Yes = 1)	8.90 (3.91, 20.28)	1.723	
	Sex (Male = 0, Female = 1)	0.21 (0.09, 0.48)	1.026	
	Diagnostic in Axis V (No = 0, Yes = 1)	0.66 (0.50, 0.89)	1.751	

VIF: Variance Inflation Factor. * VIF gives k-1 VIF values, where k is the number of values of a certain variable. In non-dichotomous variables, VIF gives more than one value. In these cases, we report the largest VIF value.

## Data Availability

Individual data are protected by European and Spanish Data Protection legislation and thus are not shareable.
